# A Case of Punctate Inner Choroidopathy in the Fellow Eye Followed by the Development of Solitary Punctate Chorioretinitis With Choroidal Neovascularization

**DOI:** 10.7759/cureus.116620

**Published:** 2026-09-21

**Authors:** Atsuki Fukushima, Tomoki Shirakami, Keigo Murata, Hitoshi Tabuchi

**Affiliations:** 1 Ophthalmology, Tsukazaki Hospital, Himeji, JPN; 2 Ophthalmology, Hiroshima University, Hiroshima, JPN

**Keywords:** choroidal neovascularization, multimodal imaging, punctate inner choroidopathy, solitary punctate chorioretinitis, white dot syndrome

## Abstract

We present the clinical course of a patient who developed punctate inner choroidopathy (PIC) followed by solitary punctate chorioretinitis (SPC), and subsequently developed choroidal neovascularization (CNV) after a prolonged period. We also discuss the importance of examination and diagnosis using multimodal imaging, as well as the significance of follow-up. A 35-year-old woman with high myopia presented with a one-week history of central visual disturbance in her left eye. Examination revealed multiple white dots in the left macula, but none in the right eye. Based on multimodal imaging findings typically for PIC, she was diagnosed with PIC and placed under observation. Three years after the initial visit, a solitary white dot appeared superonasal to the fovea in the right macula, leading to a diagnosis of SPC based on multimodal imaging. Although findings in both eyes gradually worsened thereafter, no CNV was observed. Thirteen and a half years after the onset of right eye findings, she presented with a complaint of rapid vision loss, and CNV was detected in the right eye; no CNV was observed in the left eye. Based on these results, the CNV was attributed to the SPC in the right eye, and treatment involving intravitreal anti-vascular endothelial growth factor agent injections and sub-Tenon’s injections of triamcinolone acetonide was initiated. In this case, CNV developed 13 years after the confirmation of SPC. Thus, for cases of unilateral PIC, it is essential to bear in mind the potential for developing SPC and the subsequent risk of CNV arising from SPC over the long term, and to conduct regular evaluations using multimodal imaging.

## Introduction

Punctate inner choroidopathy (PIC) was first reported by Watzke et al. in 1984 as a subtype of the white dot syndromes, a group of disorders possibly thought to involve autoimmune mechanisms [1]. Regarding prevalence, its incidence has been estimated at 0.4 cases per 1,000,000 per year [2]. The disease primarily affects young, myopic women, who often present with complaints such as reduced visual acuity, photopsia, and central scotoma. Given the suspected role of autoimmunity in its pathogenesis, PIC is considered a key differential diagnosis when classifying forms of uveitis. Consequently, diagnosing the condition requires ruling out other types of uveitis [3]. Systemic tests, such as blood analysis, are used to exclude conditions such as sarcoidosis, tuberculosis, syphilis, toxoplasmosis, and histoplasmosis. Once these have been ruled out, findings consistent with PIC are evaluated using modalities such as color fundus photography, optical coherence tomography (OCT), and fundus fluorescein angiography. While PIC can be appropriately diagnosed via color fundus photography, OCT and fundus fluorescein angiography are employed to assess lesion activity; OCT, in particular, is considered useful for evaluating inflammatory activity [4].

While PIC is characterized by multiple lesions, a distinct disease concept known as solitary punctate chorioretinitis (SPC) has been proposed based on reports of 12 cases presenting with a solitary lesion resembling the individual lesions of PIC [5]. SPC may underlie certain cases of focal choroidal excavation and idiopathic choroidal neovascularization (CNV) that were previously classified as unclassifiable. Given the potential involvement of an autoimmune response in the pathogenesis of PIC, the condition is reported to be predominantly bilateral, albeit often with asymmetry between the eyes [6]; however, unilateral cases have also been documented (e.g., two out of eight cases [7]). Although the association of SPC with CNV is recognized, there is a report of an SPC case followed without treatment for 91 months, during which the patient exhibited fluctuating CNV activity [8]. We report a case in which the patient developed PIC in one eye and SPC in the other 3.5 years later. While no CNV was observed in the PIC eye, CNV developed in the SPC eye 13 years after the initial detection of SPC findings.

## Case presentation

A 35-year-old woman presented with a one-week history of impaired central vision in her left eye. She exhibited high myopia, with refractive errors of -12 diopters (right) and -10.5 diopters (left) and axial lengths of 29.91 mm (right) and 28.29 mm (left). Best-corrected visual acuity was 1.5 in the right eye and 0.7 in the left eye, and intraocular pressure was 12 mmHg (right) and 11 mmHg (left). Multiple white dots were observed in the left macula but not in the right eye (Figures 1a, 1e). Fluorescein angiography of the right eye revealed a patchy area of hypofluorescence, persisting in both early and late phases, located slightly superior to the fovea (Figures 1b, 1c). In the left eye, corresponding to the areas where white spots were seen on color fundus photography, a patchy lesion was observed showing early-phase central hypofluorescence and peripheral hyperfluorescence (Figure 1f). In the late phase, the entire lesion became hyperfluorescent, accompanied by mild dye leakage (Figure 1g). OCT showed no abnormalities in the right eye (Figure 1d), whereas the left eye exhibited findings of outer retinal tissue, including the ellipsoid zone (EZ), being pulled toward the retinal pigment epithelium (Figure 1h).

**Figure 1:**
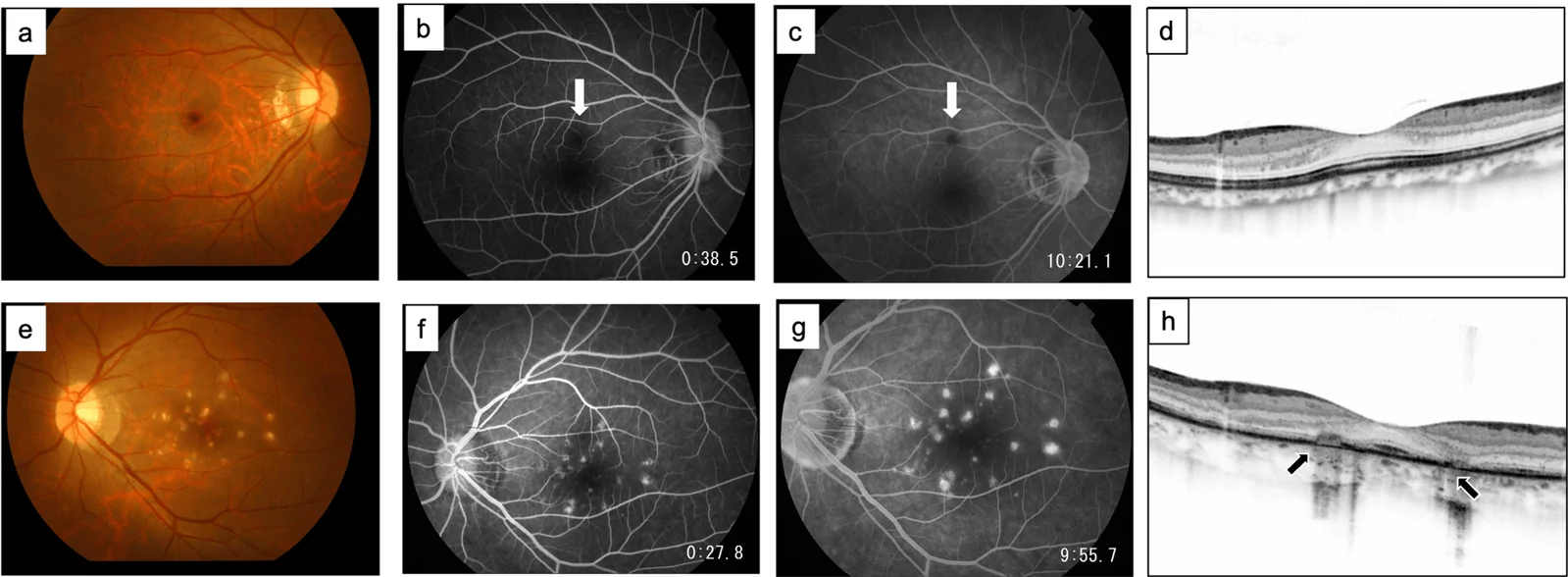
Multimodal fundus imaging at the initial visit. Color fundus photographs (right eye: a, left eye: e), fluorescein angiography (right eye early phase: b, right eye late phase: c; left eye early phase: f, left eye late phase: g), and optical coherence tomography (OCT) (right eye: d, left eye: h). Color fundus imaging revealed numerous white spots in the left macular region (e), whereas none were observed in the right eye (a). Fluorescein angiography of the right eye showed a patchy area of hypofluorescence, present in both early and late phases, located slightly superior to the fovea (b, c; white arrows). In the left eye, corresponding to the areas where white spots were seen on color fundus imaging, patchy areas were observed exhibiting central hypofluorescence and peripheral hyperfluorescence during the early phase (f). In the late phase, these areas became entirely hyperfluorescent, with mild dye leakage observed (g). OCT showed no abnormalities in the right eye (d), whereas in the left eye, findings indicated that the outer retinal layers, including the ellipsoid zone, were being pulled toward the retinal pigment epithelium (h; black arrow).

Based on these findings, the patient was diagnosed with PIC, a subtype of white dot syndrome, and placed under observation. Three and a half years after the initial visit, a solitary white dot appeared in the right eye, located slightly superotemporal to the fovea (Figure 2a). In the left eye, degeneration of the lesions progressed, with observed enlargement of the lesions and the development of pigmentation (Figure 2e). Fluorescein angiography revealed hyperfluorescence in the right eye corresponding to the solitary white dot (Figure 2b). In the left eye, findings showed hypofluorescence at the centers and hyperfluorescence at the margins of the individual lesions (Figure 2f). Indocyanine green angiography showed hypofluorescence at the site of the solitary white dot in both the early (Figure 2c) and late phases (Figure 2d). OCT revealed no obvious abnormalities in the right eye (Figure 2g), whereas in the left eye, there was a more distinct finding of outer retinal tissue being pulled toward the retinal pigment epithelium (Figure 2h).

**Figure 2:**
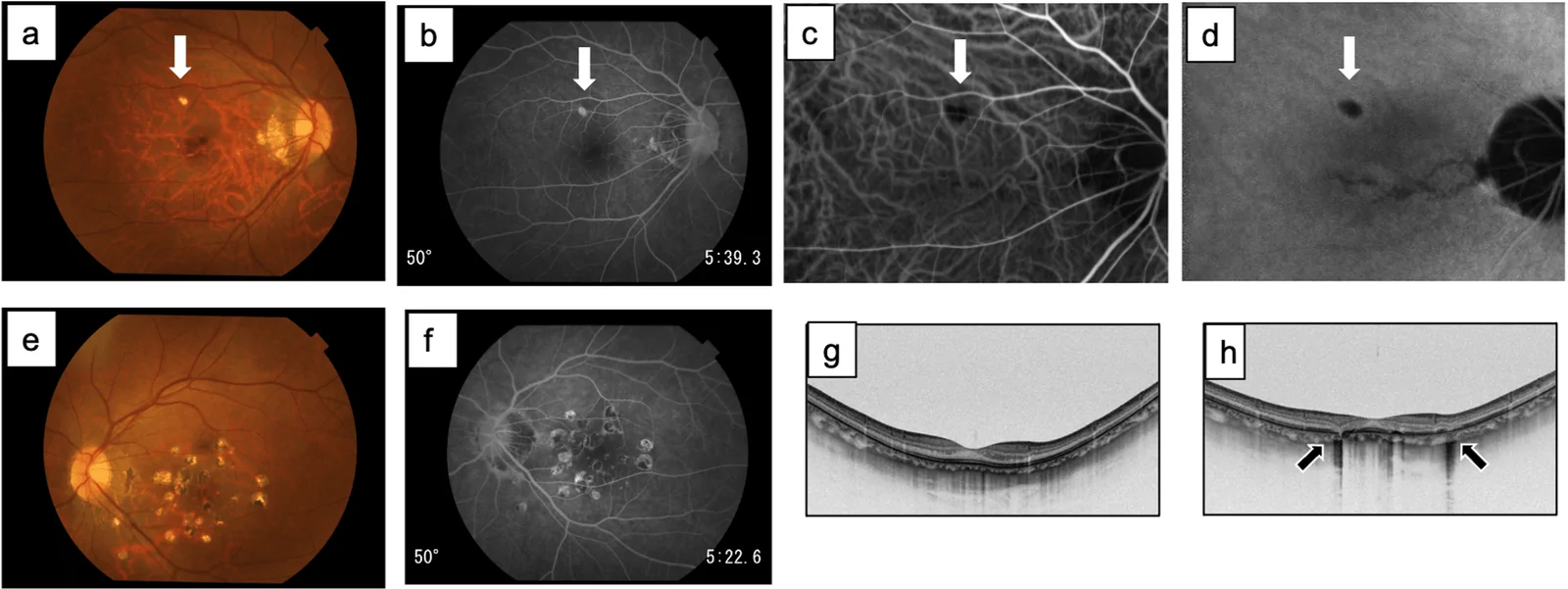
Multimodal fundus imaging 3.5 years after the initial visit. Color fundus photographs (right eye: a, left eye: e), fluorescein angiography (right eye: b, left eye: f), indocyanine green angiography (right eye early phase: c, right eye late phase: d), and optical coherence tomography (OCT) (right eye: g, left eye: h). Color fundus imaging revealed the appearance of a solitary white dot slightly superotemporal to the fovea in the right eye (a, white arrow). In the left eye, degeneration of the lesions had progressed, with observed lesion enlargement and pigmentation (e). Fluorescein angiography showed hyperfluorescence in the right eye corresponding to the solitary white dot (b, white arrow). In the left eye, findings of central hypofluorescence and peripheral hyperfluorescence were observed corresponding to the individual lesions (f). Indocyanine green angiography revealed hypofluorescence in both the early phase (c, white arrow) and late phase (d, white arrow) corresponding to the solitary white dot in the right eye. OCT showed no obvious abnormalities in the right eye (g), whereas in the left eye, the finding of outer retinal tissue being pulled toward the retinal pigment epithelium layer became more distinct (h, black arrow).

Three years later, the solitary white dot in the right eye had enlarged slightly, though the number of dots remained unchanged (Figure 3a). Pigmentation in the left eye had progressed (Figure 3c). OCT showed no abnormalities in the right eye (Figure 3b), but damage to the EZ in the left eye had become more extensive (Figure 3d).

**Figure 3:**
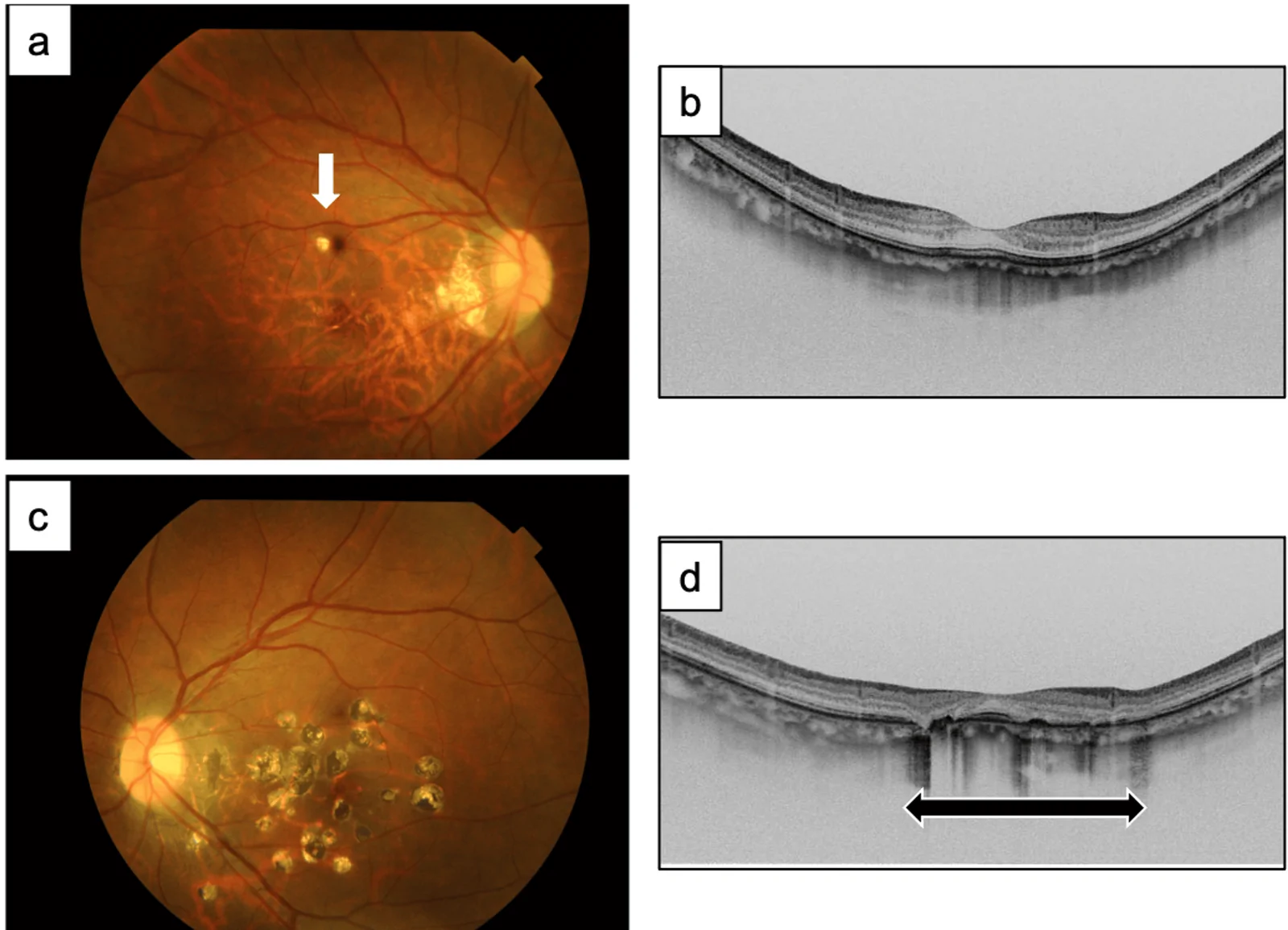
Multimodal fundus imaging 6.5 years after the initial visit. Color fundus photographs (right eye: a, left eye: c) and optical coherence tomography (OCT) images (right eye: b, left eye: d). The area of the solitary white spot in the right eye had expanded slightly, but the number of spots remained unchanged (a, white arrow). Pigmentation in the left eye had progressed (c). No abnormalities were observed on OCT in the right eye (b), whereas the disruption of the ellipsoid zone in the left eye had become more extensive (d, double-headed black arrows).

Eight years later, the area of the solitary white spot in the right eye (Figure 4a) and the area of pigmentation in the left eye (Figure 4c) had gradually enlarged, although the number of white spots in the right eye had not increased (Figure 4a). OCT revealed no notable abnormalities in the right fovea (Figure 4b), whereas damage to the outer retinal layers in the left eye had progressed further (Figure 4d).

**Figure 4:**
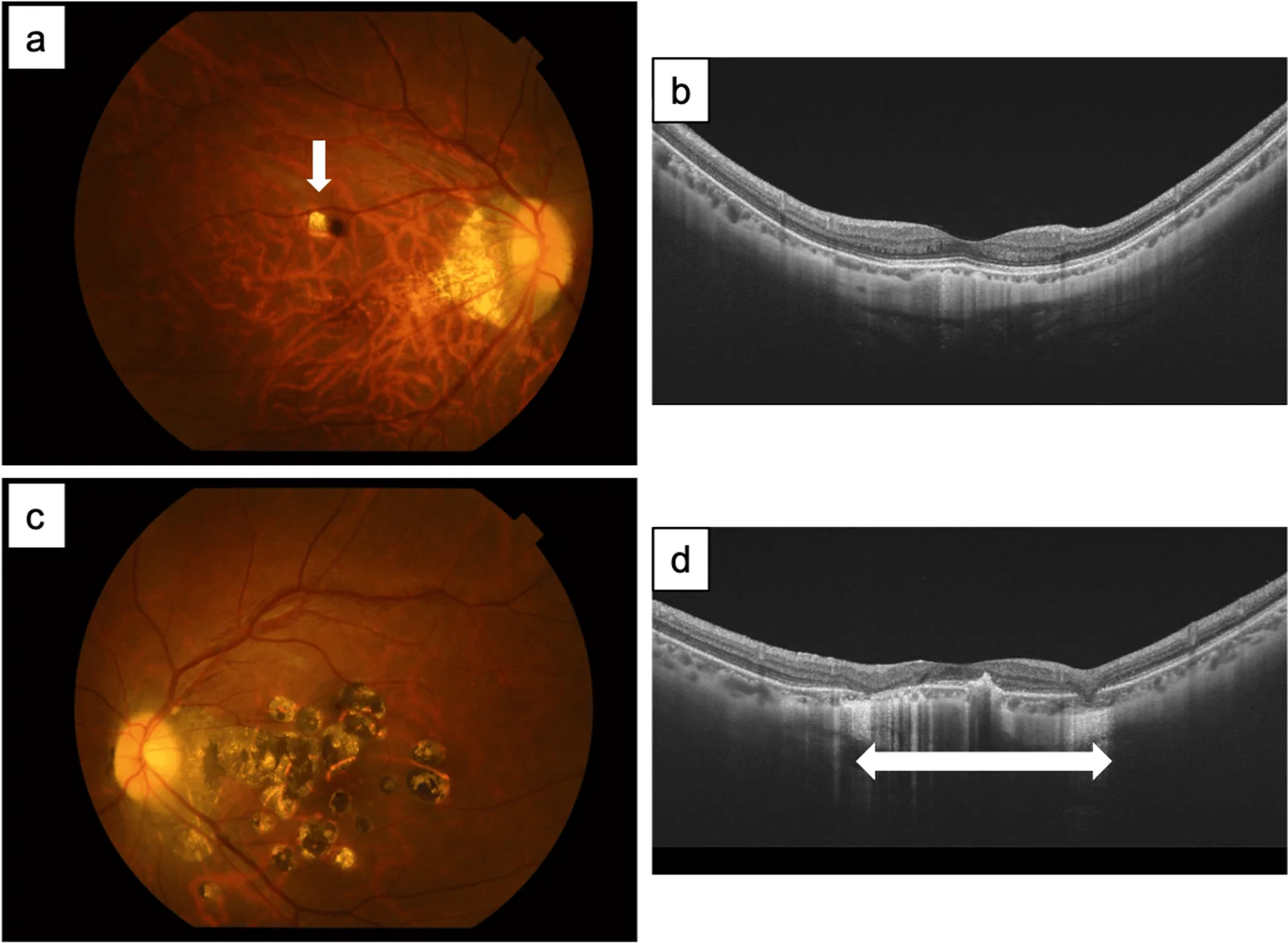
Multimodal fundus imaging 11.5 years after the initial visit. Color fundus photographs (right eye: a; left eye: c) and optical coherence tomography (OCT) images (right eye: b; left eye: d). The area of the solitary white spot in the right eye (a, white arrow) and the pigmentation in the left eye (c) were gradually enlarging, although the number of white spots in the right eye had not increased (a, white arrow). OCT showed no notable abnormalities in the fovea of the right eye (b), whereas damage to the outer retinal layers in the left eye had progressed further (d, double-headed white arrow).

Follow-up examinations were continued every six months thereafter, with no significant worsening observed. The patient returned for re-examination 16.5 years after the initial visit (13 years after the onset of symptoms in the right eye), presenting with a chief complaint of visual impairment in the right eye that had persisted for two months and a sudden decline in vision starting the previous day. The area of the solitary white spot in the right eye (Figure 5a) and the area of pigmentation in the left eye had expanded further (Figure 5e). Fluorescein angiography showed that the solitary white spot in the right eye exhibited hypofluorescence in the early phase (Figure 5b), while the periphery showed ring-like hyperfluorescence in the late phase (Figure 5c). Regarding the right fovea, no notable abnormalities were observed in the early phase (Figure 5b), but fluorescein leakage was detected in the late phase (Figure 5c). In the left eye, ring-like hyperfluorescence was observed around each white lesion from the early phase (Figure 5f). In the late phase, tissue staining was observed at each lesion site, but no leakage of fluorescent dye, including at the fovea, was detected (Figure 5g). OCT imaging of the right eye revealed a disruption in the retinal pigment epithelium and the invasion of choroidal tissue into the retina (indicating CNV) (Figure 5d). The left eye showed progression of degeneration without invasion of choroidal tissue into the retina (Figure 5h). Additionally, an epiretinal membrane (ERM) was noted only in the right eye (Figure 5d).

**Figure 5:**
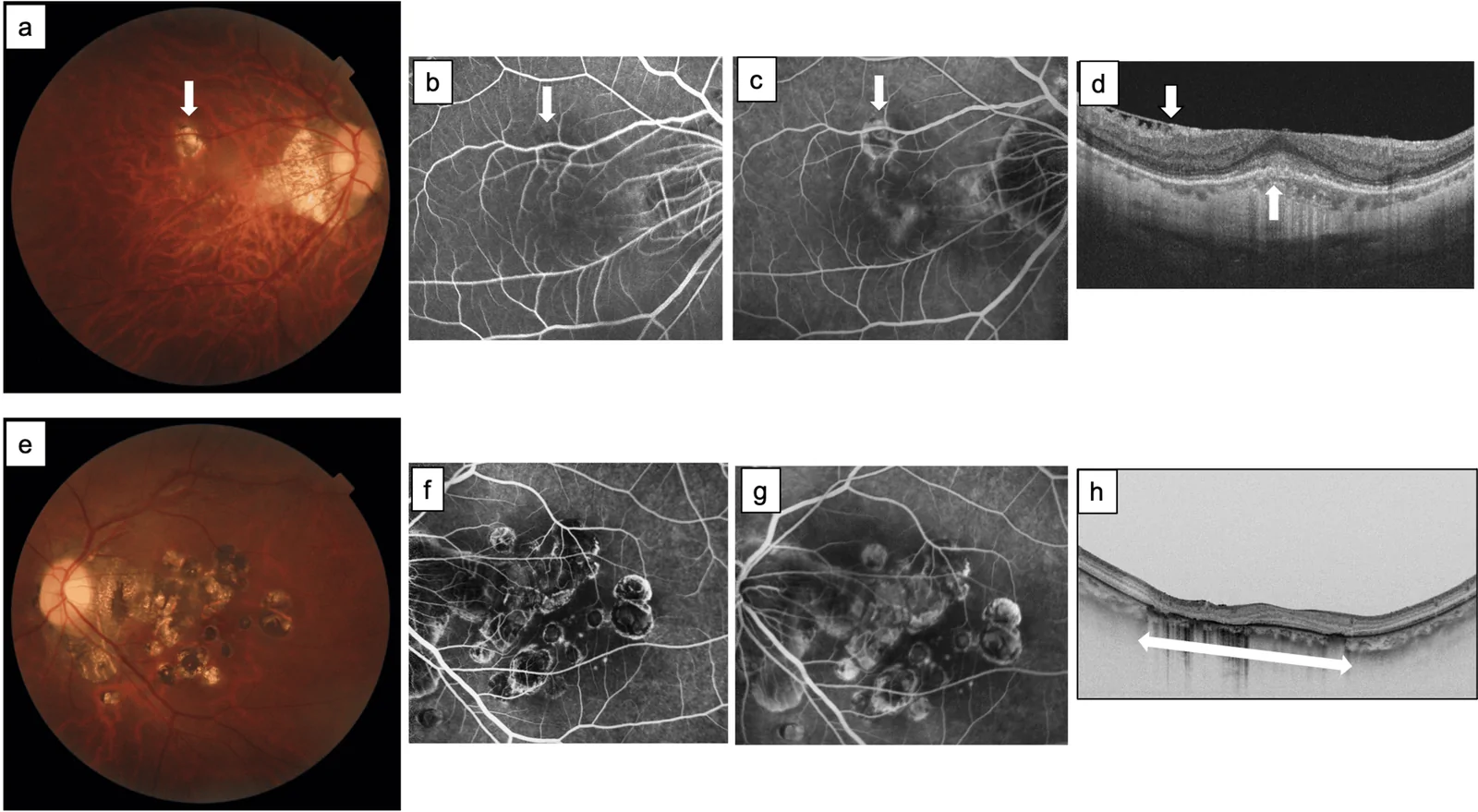
Multimodal fundus imaging 16.5 years after the initial visit. Color fundus photographs (right eye: a, left eye: e), fluorescein angiography images (right eye early phase: b, right eye late phase: c, left eye early phase: f, left eye late phase: g), and optical coherence tomography (OCT) images (right eye: d, left eye: h). The patient returned for a follow-up visit 13 years after the onset of symptoms in the right eye. Color fundus photography revealed that a solitary white spot in the right eye was slightly enlarged (a, white arrow), while the area of pigmentation in the left eye had further expanded (e). Fluorescein angiography showed hypofluorescence at the site of the solitary white spot in the right eye during the early phase (b, white arrow) and a ring-like area of hyperfluorescence at the periphery during the late phase (c, white arrow). Regarding the fovea of the right eye, no notable abnormalities were observed in the early phase (b), whereas fluorescein leakage was noted in the late phase (c, white dashed circle). In the left eye, ring-like hyperfluorescence around each white lesion was observed from the early phase (f). In the late phase, tissue staining of the lesions was observed, but no fluorescein leakage, including at the fovea, was detected (g). OCT revealed a disruption of the retinal pigment epithelium and the invasion of choroidal tissue into the retina (choroidal neovascularization) in the right eye (d, white upward arrow); in the left eye, although degeneration progressed, no invasion of choroidal tissue into the retina was observed (h, double white arrows). An epiretinal membrane was noted only in the right eye (d, white down arrow).

OCT evaluation of the isolated white spot in the right eye revealed a heterogeneous lesion, exhibiting moderate-to-high reflectivity and irregular outer retinal layers with indistinct boundaries, in both the transverse (Figure 6a) and longitudinal (Figure 6b) cross-sections.

**Figure 6:**
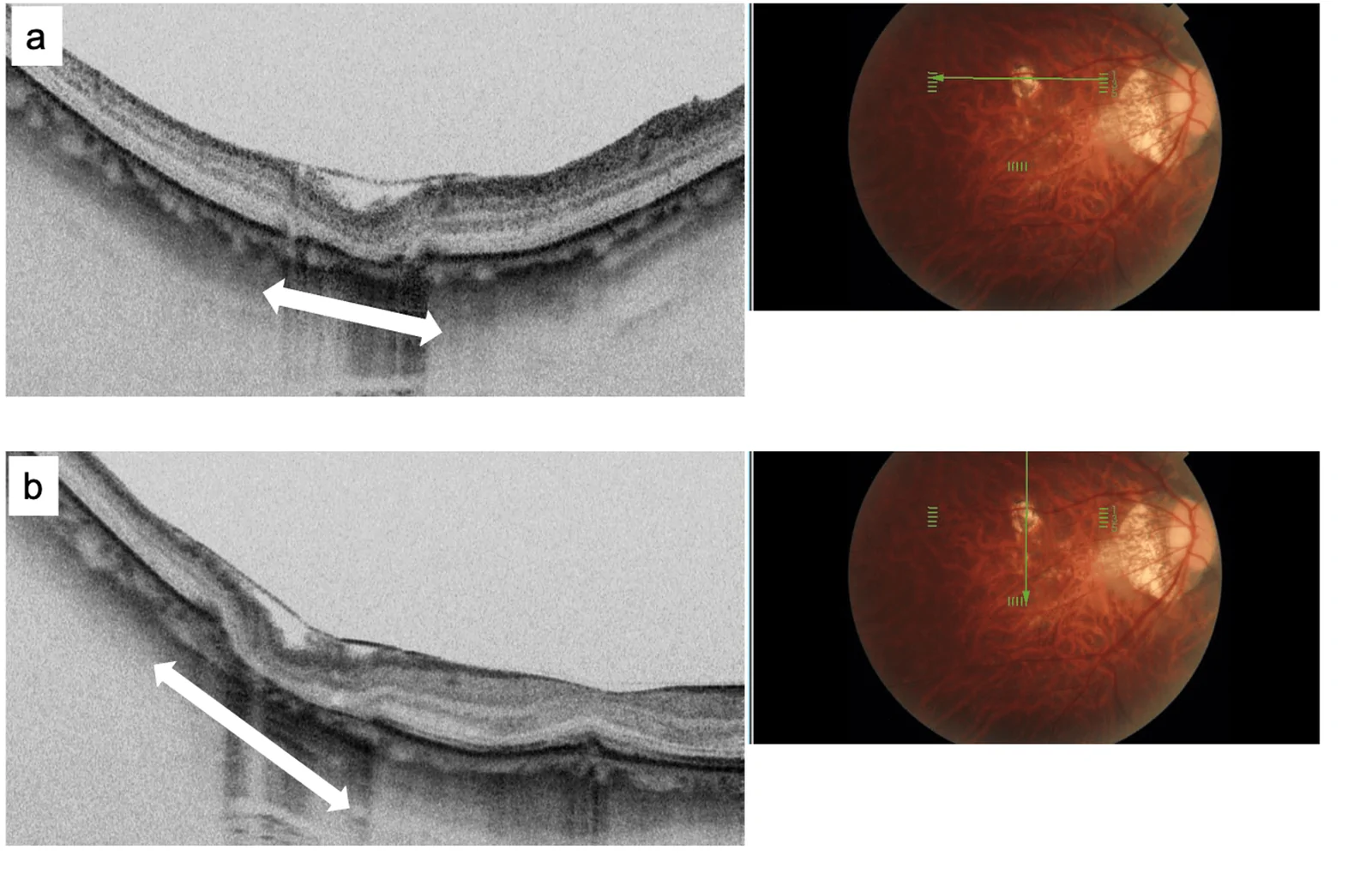
Optical coherence tomography of the solitary white spot in the right eye 16.5 years after the initial visit. (a) Horizontal section; (b) vertical section. In both the horizontal section (a, double-headed white arrows) and the vertical section (b, double-headed white arrows), a heterogeneous lesion with moderate-to-high reflectivity, indistinct boundaries, and irregularity of the outer retinal layers was observed.

Follow-up was initiated involving intravitreal injection of an anti-vascular endothelial growth factor agent and sub-Tenon’s injection of triamcinolone acetonide, because PIC is considered to be mediated by an autoimmune reaction [1].

## Discussion

First, we present the evidence supporting the diagnosis of SPC in this case. A diagnosis of SPC is made when characteristic findings are observed via standardized multimodal imaging, as reported previously [9]. Fundus photography revealed a solitary, punctate, yellowish-white lesion in the area corresponding to the outer retina and choroid (Figure 2a). Fluorescein angiography showed hyperfluorescence and staining (Figure 2b). Indocyanine green angiography demonstrated hypofluorescence in both the early and late phases (Figures 2c, 2d). Swept-source OCT imaging revealed a solitary, heterogeneous lesion of moderate-to-high reflectivity with indistinct boundaries that disrupted the outer retinal structure (Figure 6). Given that all findings are consistent with previously reported multimodal imaging characteristics [8] and that PIC was observed in the fellow eye, a diagnosis of SPC is appropriate.

Next, it is necessary to differentiate the condition from myopic choroidal neovascularization (mCNV). In 2015, the Pathologic Myopia Study Group proposed a classification system subdividing myopic maculopathy into the following five categories: (0) no maculopathy, (1) tessellated fundus, (2) diffuse chorioretinal atrophy, (3) patchy chorioretinal atrophy, and (4) macular atrophy. Lacquer cracks and mCNV were identified as “plus lesions.” Pathologic myopia is defined as myopic maculopathy category 2 or higher accompanied by plus lesions or posterior staphyloma [10]. Although the present case involves high myopia, the findings are limited to a tessellated fundus and do not meet the criteria for pathologic myopia. While myopic changes certainly play a role in the development of CNV, the condition of our case is not considered to be “myopic choroidal neovascularization” in the strict sense.

A large-scale cohort study on PIC involving 203 patients (318 eyes) reported that bilateral lesions were present in 115 (56.7%) patients at the initial visit, and choroidal neovascular membranes (CNVMs) were observed in 152 (47.8%) eyes. During a median follow-up period of 8.4 years, the development of new CNVM was also confirmed; CNVM was observed in 33.5% of eyes affected by PIC and in 4.3% of initially unaffected eyes, totaling 58 eyes. An increased risk of CNVM development was associated with the presence of CNVM in the fellow eye (p < 0.0005; hazard ratio (HR) = 2.73). Additionally, prior treatment with oral corticosteroids was associated with a halving of the risk of CNVM development (p = 0.035; HR = 0.45) [11], suggesting the involvement of immune responses, specifically autoimmune responses, in the pathogenesis of PIC.

On the other hand, there are reports of CNV occurring in 16.67% [5] and 29.4% [12] of SPC cases. Although the incidence of CNV in SPC appears to be lower than in PIC, it is highly likely that both diseases share the same underlying pathogenesis. Consequently, the higher probability of CNV development in PIC may be attributed to the greater number of lesions, which results in a more extensive area of involvement. In any case, given the limited number of reports regarding CNV in SPC, further accumulation of clinical cases is necessary.

A notable feature of this case is that, while 16.5 years have passed since the onset in the PIC eye, no CNV has developed; conversely, CNV was observed in the SPC eye after a prolonged period of 13 years. Although high myopia is present in both eyes, the longer axial length of the right eye compared to the left may contribute to macular traction [13]. It has been reported that exudative changes can alter the vitreoretinal interface and lead to the development of an ERM [14]. In this case, too, it is likely that exudative changes were present gradually even before the CNV became distinct. Furthermore, the potential involvement of ERM in macular traction has been documented [15]. Although our results provide no evidence that the ERM is exerting traction on the fovea, in our case, the ERM may play a role in macular traction of the right eye (Figure 5d). These factors may have contributed to the development of CNV in the SPC eye. It is hypothesized that the combination of long-standing, low-grade inflammation and the aforementioned conditions compromised the structural integrity of the outer retina, ultimately leading to CNV. Crucially, given that the risk of CNV development in SPC appears to increase with the duration of the condition, long-term, consistent follow-up is essential.

## Conclusions

PIC is characterized by multiple lesions. Recently, a distinct disease concept known as SPC has been proposed based on reports of 12 cases presenting with a solitary lesion resembling the individual lesions of PIC. Here, we report a case in which the patient developed PIC in one eye and SPC in the other 3.5 years later. While no CNV was observed in the PIC eye, CNV developed in the SPC eye 13 years after the initial detection of SPC findings. These findings highlight the need to consider the potential for developing SPC in cases of unilateral PIC, as well as the risk of subsequent CNV development in SPC, and underscore the importance of regular monitoring using multimodal imaging.
